# Sound as a bell: a deep learning approach for health status classification through speech acoustic biomarkers

**DOI:** 10.1186/s13020-024-00973-3

**Published:** 2024-07-24

**Authors:** Yanbing Wang, Haiyan Wang, Zhuoxuan Li, Haoran Zhang, Liwen Yang, Jiarui Li, Zixiang Tang, Shujuan Hou, Qi Wang

**Affiliations:** 1 School of Traditional Chinese Medicine, Beijing University of Chinese Medicine, Beijing, 100029 China; 2 School of Management, Beijing University of Chinese Medicine, Beijing, 100029 China; 3 National Institute of TCM Constitution and Preventive Medicine, Beijing University of Chinese Medicine, Beijing, 100029 China

**Keywords:** Speech analysis, Acoustic parameters, Deep learning, Body constitution, Subhealth, Traditional Chinese Medicine

## Abstract

**Background:**

Human health is a complex, dynamic concept encompassing a spectrum of states influenced by genetic, environmental, physiological, and psychological factors. Traditional Chinese Medicine categorizes health into nine body constitutional types, each reflecting unique balances or imbalances in vital energies, influencing physical, mental, and emotional states. Advances in machine learning models offer promising avenues for diagnosing conditions like Alzheimer's, dementia, and respiratory diseases by analyzing speech patterns, enabling complementary non-invasive disease diagnosis. The study aims to use speech audio to identify subhealth populations characterized by unbalanced constitution types.

**Methods:**

Participants, aged 18–45, were selected from the Acoustic Study of Health. Audio recordings were collected using ATR2500X-USB microphones and Praat software. Exclusion criteria included recent illness, dental issues, and specific medical histories. The audio data were preprocessed to Mel-frequency cepstral coefficients (MFCCs) for model training. Three deep learning models—1-Dimensional Convolution Network (Conv1D), 2-Dimensional Convolution Network (Conv2D), and Long Short-Term Memory (LSTM)—were implemented using Python to classify health status. Saliency maps were generated to provide model explainability.

**Results:**

The study used 1,378 recordings from balanced (healthy) and 1,413 from unbalanced (subhealth) types. The Conv1D model achieved a training accuracy of 91.91% and validation accuracy of 84.19%. The Conv2D model had 96.19% training accuracy and 84.93% validation accuracy. The LSTM model showed 92.79% training accuracy and 87.13% validation accuracy, with early signs of overfitting. AUC scores were 0.92 and 0.94 (Conv1D), 0.99 (Conv2D), and 0.97 (LSTM). All models demonstrated robust performance, with Conv2D excelling in discrimination accuracy.

**Conclusions:**

The deep learning classification of human speech audio for health status using body constitution types showed promising results with Conv1D, Conv2D, and LSTM models. Analysis of ROC curves, training accuracy, and validation accuracy showed all models robustly distinguished between balanced and unbalanced constitution types. Conv2D excelled with good accuracy, while Conv1D and LSTM also performed well, affirming their reliability. The study integrates constitution theory and deep learning technologies to classify subhealth populations using noninvasive approach, thereby promoting personalized medicine and early intervention strategies.

## Background

Human health is a nuanced concept that extends beyond a simple dichotomy of being either healthy or diseased. Instead, it encompasses a diverse range of states and variations along a continuum. This landscape of health is characterized by its complexity, dynamic nature, and multifaceted interplay of genetic and environmental factors [1], as well as factors related to physiology and psychology [2].

Individuals experience a variety of physical, mental, and emotional states, each with its own nuances and characteristics. These states can range from optimal health to mild discomfort, occasional illness, chronic conditions, and varying degrees of wellness or unwellness. Health is not static but rather dynamic, influenced by factors such as lifestyle choices, environmental exposures, genetic predispositions, and social determinants. Socioeconomic status, access to healthcare, and support networks also significantly impact health outcomes and experiences [3–5]. Taking a holistic perspective on wellness acknowledges that health is more than just the absence of disease; it encompasses overall well-being and resilience.

In Traditional Chinese Medicine (TCM), the nine body constitutional types are viewed as dynamic states of health, each representing a unique balance or imbalance within the body's vital energies: Balanced constitution (BC), Qi Deficiency Constitution (QDC), Yang Deficiency Constitution (YDC), Yin Deficiency Constitution (YnDC), Phelgm-dampness constitution (PDC), Dampness-heat constitution (DHC), Blood stasis constitution (BSC), Qi Stagnation Constitution (QSC), and Special Diathesis Constitution (SDC) [6]. These constitutional types reflect not only physical traits but also psychological, emotional and mental characteristics, offering insight into an individual's overall well-being. Correlative relationships between constitution and diseases indicates an association between an individual's physiological makeup or health condition and the likelihood or manifestation of various illnesses or health issues [7]. For example, individuals with YnDC may experience fluctuations in body temperature and heightened anxiety, reflecting a shortage of Yin energies. Similarly, those with YDC may exhibit signs of weakness and fatigue, indicating a deficiency in Yang energies. Individuals with a PDC constitution face significantly elevated risks of obesity, metabolic syndrome, hypertension, and diabetes compared to those with a balanced body constitution type [8]. Moreover, recent research has shown that significant differences in taxonomic features exist between DHC and balanced cohorts, with distinct compositions at the phylum, family, and genus levels, including notable variations in Enterococcaceae, Pasteurellaceae, Subdoligranulum, E. hallii, Haemophilus, and Enterococcus [9]. These constitutional types are not fixed categories but rather dynamic states that can evolve over time in response to various internal and external factors. Irregular, unhealthy lifestyles causing dysrhythmic circadian can contribute to the development of a phlegm-dampness constitution [10]. By understanding and addressing these constitutional imbalances through TCM therapies such as acupuncture, herbal medicine, and lifestyle adjustments, individuals can work towards restoring harmony and promoting optimal health. Questionnaire instruments, both long and short form, have been developed and validated to measure the constitution types [11].

The balanced type is considered a state of harmonious balance in which the body's vital energies, or Qi, are in equilibrium, leading to overall good health. The remaining eight types denote different types and levels of imbalance or disharmony in the body's Qi, leading to physical, mental, and emotional symptoms linked to suboptimal health.

Individuals classified as balanced are seen as healthy and resilient, with few symptoms of discomfort or imbalances compared to those classified into the other constitutional types. Meanwhile, individuals with the other eight unbalanced types may experience a range of symptoms and health challenges due to imbalances in their Qi, indicating a state of suboptimal health. Recognizing these constitutional types and their associated imbalances, TCM practitioners can tailor treatment approaches to address the specific needs of each individual, aiming to restore harmony and promote overall well-being.

Human speech voice production is a complex process that involves the coordinated efforts of multiple organs within the respiratory, vocal, and neurological systems. These organs include the brain, the lungs, vocal cords, larynx, pharynx, mouth, tongue, and lips. The lungs provide the necessary airflow and pressure for speech, while the vocal cords, located in the larynx, vibrate to produce sound. The larynx manipulates the vocal cords to generate different pitches and volumes. As the sound travels through the pharynx, it acts as a resonating chamber, contributing to the overall quality of the voice. Further shaping of the sound occurs in the mouth, where the tongue and lips articulate different speech sounds by controlling the size and position of the oral cavity. Each of these organs plays a vital role in the production of speech sounds, with their coordinated movements allowing for the precise articulation of spoken language. Dysfunction or impairment in any of these organs can affect speech production and may lead to speech disorders or difficulties.

Auditory diagnosis, known as Tingzhen in TCM, is an important component of four diagnostic methods in TCM diagnosis. This diagnostic method involves listening to sounds produced by the body, such as the heartbeat, breath sounds, sounds within the abdomen, and more importantly human speech sound, to gain insights into the patient's internal health condition. TCM practitioners believe that the sounds heard during listening diagnosis can provide valuable information about the state of the organs, Qi (vital energy) flow, and overall balance of Yin and Yang energies within the body. For example, irregularities in heart sounds may indicate issues with the cardiovascular system, while abnormal breath sounds could suggest respiratory disorders.

By integrating paralinguistic listening into the diagnostic process along with other TCM diagnostic methods such as visual inspection, palpation, and inquiry, practitioners can develop a comprehensive understanding of the patient's health condition. This holistic approach allows for the customization of treatment strategies aimed at restoring balance and promoting overall well-being. While modern medical diagnostics rely heavily on advanced imaging and laboratory tests, listening remains a valuable tool in TCM diagnosis, reflecting the tradition's emphasis on nontraumatic observation and interpretation of subtle bodily cues.

The exploration of speech acoustic features in neurodegenerative diseases such as dementia has been underway. Due to the progressive deterioration of brain regions responsible for communication and language processing, neurodegenerative diseases affecting dementia can impact a person's speech and language, thus becoming an obvious candidate in speech acoustic feature research. Machine learning classification algorithms were employed to distinguish between the Alzheimer's disease or mild cognitive impairment group and the functional cognitive disorder or healthy control group, each comprising 15 samples, achieving an accuracy rate of 80% [12, 13]. A dataset with 78 samples in each class were trained using regular machine learning methods and deep learning models to distinguish between healthy patients and Alzheimer’s patients with an accuracy of 85.4% on the test set [14]. The same dataset was analyzed using multimodal deep learning model with an accuracy of 90.00%. Voice recordings from the Framingham Heart Study using deep learning were able to detect general dementia with 74.3% accuracy using Convolutional Neural Network (CNN) model and 73.4% accuracy using Long Short-Term Memory (LSTM) model [15]. Voice data collected from 53 age-matched depression and dementia participants were analyzed using machine learning models and achieved an accuracy rate of 62.7% with unsupervised model [16]. Audio data from a previously curated dementia databank was analyzed with various machine learning and deep learning models with accuracy rates from 77 to 87% in classifying dementia patients from healthy controls [17].

Respiratory diseases are another topic of interest in biomedical acoustic research, due to their impact on lung function affecting the ability to control airflow and vocalization, thus leading to changes in voice quality and articulation. Participants with positive, recovered, and negative COVID status were able to be classified using convolutional neural network model with reported high accuracy [18]. Speech audio recordings in conjunction of cough and breathing sound were used in hybrid deep learning models to detect COVID status with Area Under the Receiver Operating Characteristic curve (AUROC) of 0.79 (0.74–0.84) [19]. Deep learning models using 76 post COVID‐19 patients and 40 healthy controls produced a classification with 85% accuracy [20].

While the apparent pathological conditions exert significant effects on vocalization via both the nervous and respiratory systems, the impact of suboptimal health (subhealth) on vocalization is expected to be more nuanced, with these subtle distinctions posing challenges for human auditory perception. While the distinction between health and disease is often clear-cut, with disease characterized by identifiable symptoms and pathology, the line between health and subhealth is less defined. Subhealth refers to a state of reduced vitality and well-being, often characterized by vague symptoms such as fatigue, low energy, and mood disturbances, which fall short of meeting the criteria for a diagnosable disease. However, subhealth can still significantly impact an individual's quality of life and predispose them to developing more serious health conditions if left unaddressed. Therefore, recognizing and addressing subhealth is crucial for maintaining overall well-being and preventing the progression to disease. The current study theorizes that through the analysis of human speech audio using deep learning algorithms, it may be possible to differentiate between individuals experiencing optimal health and those with suboptimal health. This distinction could enable early intervention strategies to prevent individuals from progressing to more severe states of illness. By leveraging advanced techniques in deep learning, we aim to build deep learning models to distinguish between subhealth and health populations.

## Methods

The aim of the study is to build deep learning models to classify healthy populations and suboptimal healthy populations. The participants are selected from Acoustic Study of Health (ASH), aiming to identify subtle patterns and characteristics in speech audio that correlate with variations in health status. Audio recordings were obtained from healthy adults 18–45 years old with balanced body constitution type, representing healthy populations, and unbalanced type, representing populations with suboptimal health.

### Exclusion criteria

Cold- or flu-like symptoms.

Missing teeth.

History of lip/jaw surgery.

Thyroidectomy.

Other respiratory-related conditions.

Neurological-related conditions.

Consumption of spicy foods within 2 h of voice recording.

### Inclusion criteria

Healthy adults between 18 and 45 years of age with no diagnosed ailments.

### Voice recording

Voice recording from participants were recorded using ATR2500X-USB microphone (Audio-technica, Tokyo, Japan). Microphone were two meters from the computer. Audios recorded using Praat (version 6.2) [21] with mono channel, 16-bit depth and 44,100 Hz in frequency. Participants’ body constitution type was screened by a questionnaire [22] and reviewed by two investigators.

Participants were asked to read a series of Chinese characters using their usual tone of voice. These characters included various vowel and consonant combinations, with sounds naturally produced from different parts of the mouth, such as the lips/teeth, middle part, and throat. Each recording was meticulously reviewed to ensure it was of high quality and free from background noise.

### Audio waves to MFCCs

**Fig. 1:**
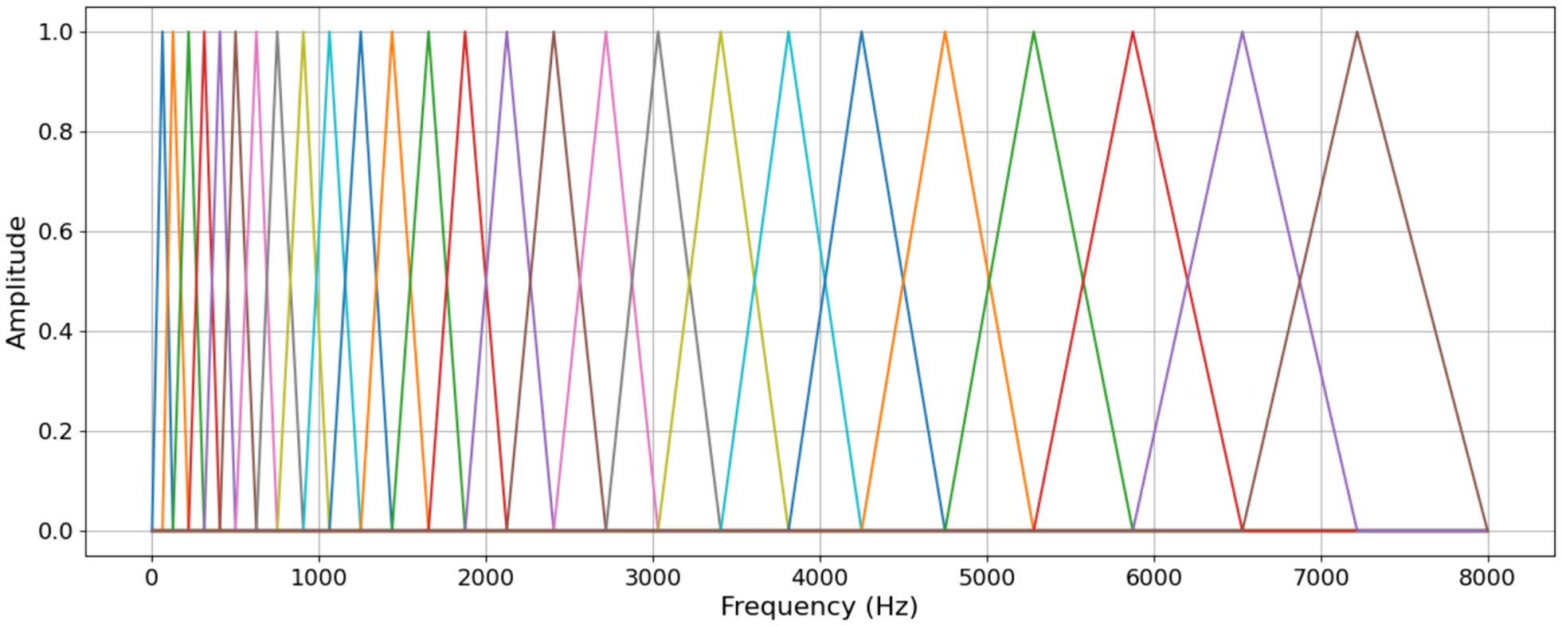
Mel-scale filter bank

A pre-emphasis filter on the signal was applied to the audio signal to amplify the high frequencies. The audio signal was then divided into short, overlapping frames (40 ms in length) to capture the time-varying nature of speech. A window function (16 ms in length) was applied to each frame to minimize spectral leakage and improve frequency resolution. The Fast Fourier Transform was used to convert each frame from the time domain to the frequency domain, resulting in a complex-valued spectrum. Mel-scale filterbank filters was applied to the spectrum to approximate the frequency selectivity of the human auditory system (Fig. 1). The filters were spaced linearly at low frequencies and logarithmically at high frequencies, according to the Mel scale. The Discrete Cosine Transform (DCT) was used to decorrelate the filterbank outputs and compress the spectral information into a smaller set of coefficients (typically 12–20). The DCT coefficients are the cepstral coefficients, and the first coefficient (C0) represents the overall energy of the frame. The current study retained the first 13 cepstral coefficients, as they were found to be the most informative for the audio classification tasks. The resulting MFCCs were used as input features for the deep learning models.

### Deep learning models

Final constitution type, either balanced type or unbalanced types, was determined using R programming language [23].

Deep learning models were implemented using Python programming language (version 3.11) with tensorflow. Before being inputted into the neural network, the audio data underwent preprocessing using Python library kapre [24] where they were initially converted into MFCCs using the Python library librosa [25]. MFCCs, a representation of the short-term power spectrum of sound, commonly used in speech and audio signal processing tasks, capture essential features of the audio signal, including frequency content, temporal dynamics, and spectral characteristics, making them suitable for audio analysis and classification tasks.

**Fig. 2:**
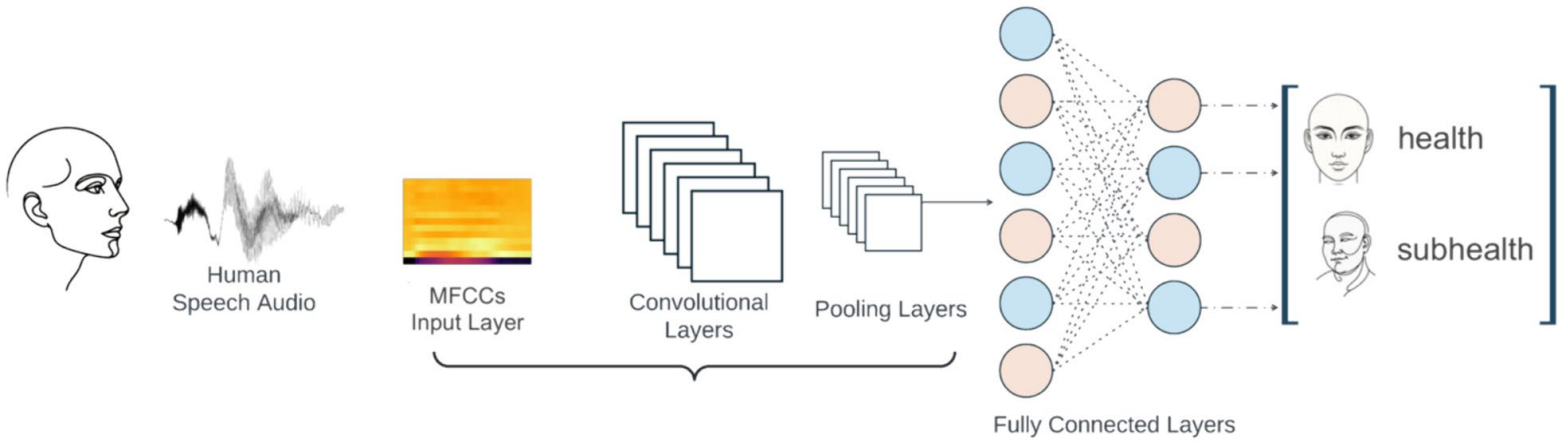
Deep learning (convolution) network architecture diagram

**Fig. 3:**
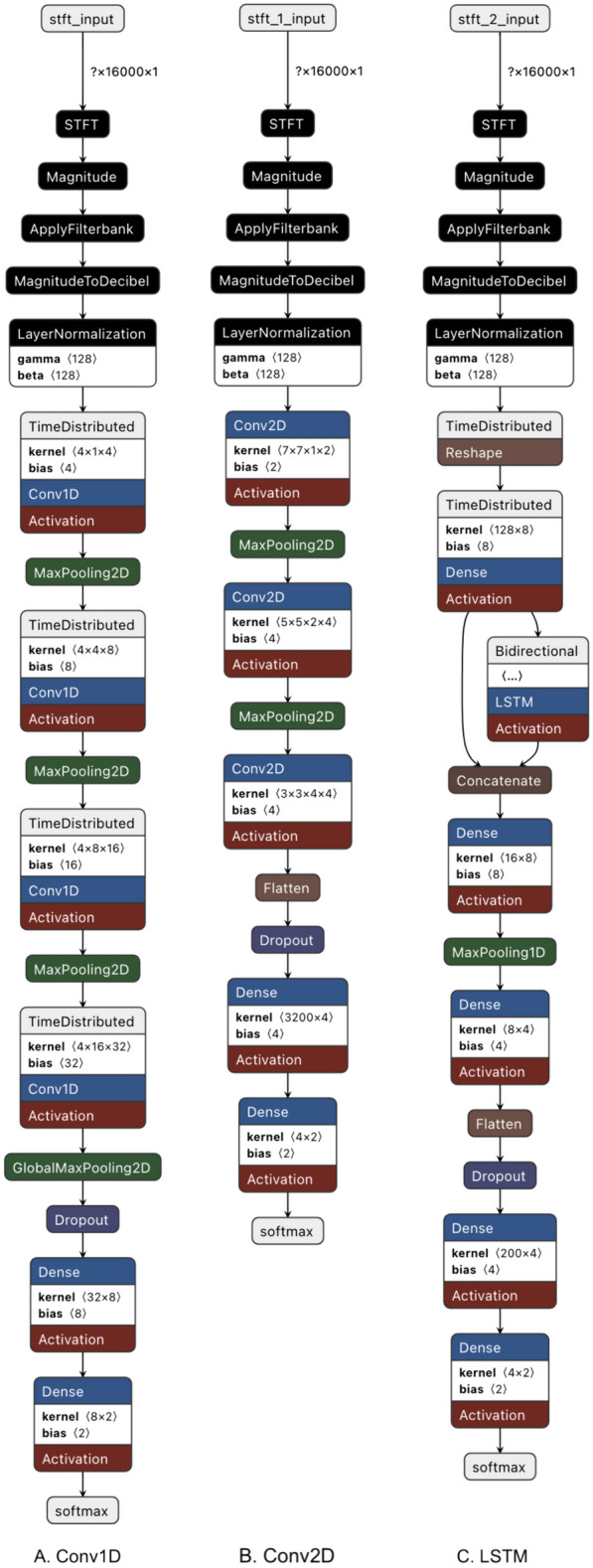
Deep learning model layers

Three different deep learning models were constructed: a 1-Dimensional Convolution (Conv1D), a 2-Dimensional Convolution (Conv2D) (Fig. 2), and a Long Short-Term Memory (LSTM) model, the first two of which are convolutional neural networks, while the last is a recurrent neural network (Fig. 3).

### Model performance metrics

Confusion matrix of the trained model is generated. True Positive (TP) is defined as balanced instances correctly predicted as balanced. True Negative (TN) is unbalanced instances correctly predicted as unbalanced. False Positive (FP) is unbalanced instances incorrectly predicted as balanced. False Negative (FN) is balanced instances incorrectly predicted as unbalanced.

*Accuracy* is the overall percentage of correctly predicted instances:

$$Accuracy = \frac{{TP + TN}}{{TP + TN + FP + FN}}$$

*Precision* is the percentage of correctly predicted “balanced” instances out of all instances predicted as “balanced.”

$$Precision = \frac{{TP}}{{TP + FP}}$$

*Recall* is the percentage of correctly predicted “balanced” instances out of all actual “balanced” instances.

$$Recall = \frac{{TP}}{{TP + FN}}$$

*F1 Score* is the harmonic mean of *precision* and *recall:*

$$F1 Score = 2 \times \frac{{Precision \times Recall}}{{Precision + Recall}}$$

*Specificity* is the percentage of correctly predicted “unbalanced” instances out of all actual “unbalanced” instances:

$$Specificity = \frac{{TN}}{{TN + FP}}$$

### Model visual explanation

To provide a visual explanation of how the model works, we generate a saliency map by watching Mel-Frequency Cepstral Coefficients (MFCCs). First, the audio signal is passed through a model that includes several preprocessing layers to convert the raw audio into MFCCs. Once the model is trained, the gradient of the model's output with respect to the MFCC input is computed. This gradient highlights parts of the MFCC features are most influential for the model's predictions, creating a saliency map that visually represents the importance of different MFCC components in the decision-making process.

## Results

A total of 1,378 audio recordings from the Balanced type (representing healthy adults) and 1,413 audio recordings from randomly chosen Unbalanced types (representing individuals with subhealth status) were used to train and validate the models.

### Comparison of audio waves and MFCCs of balanced and unbalanced constitution types

**Fig. 4:**
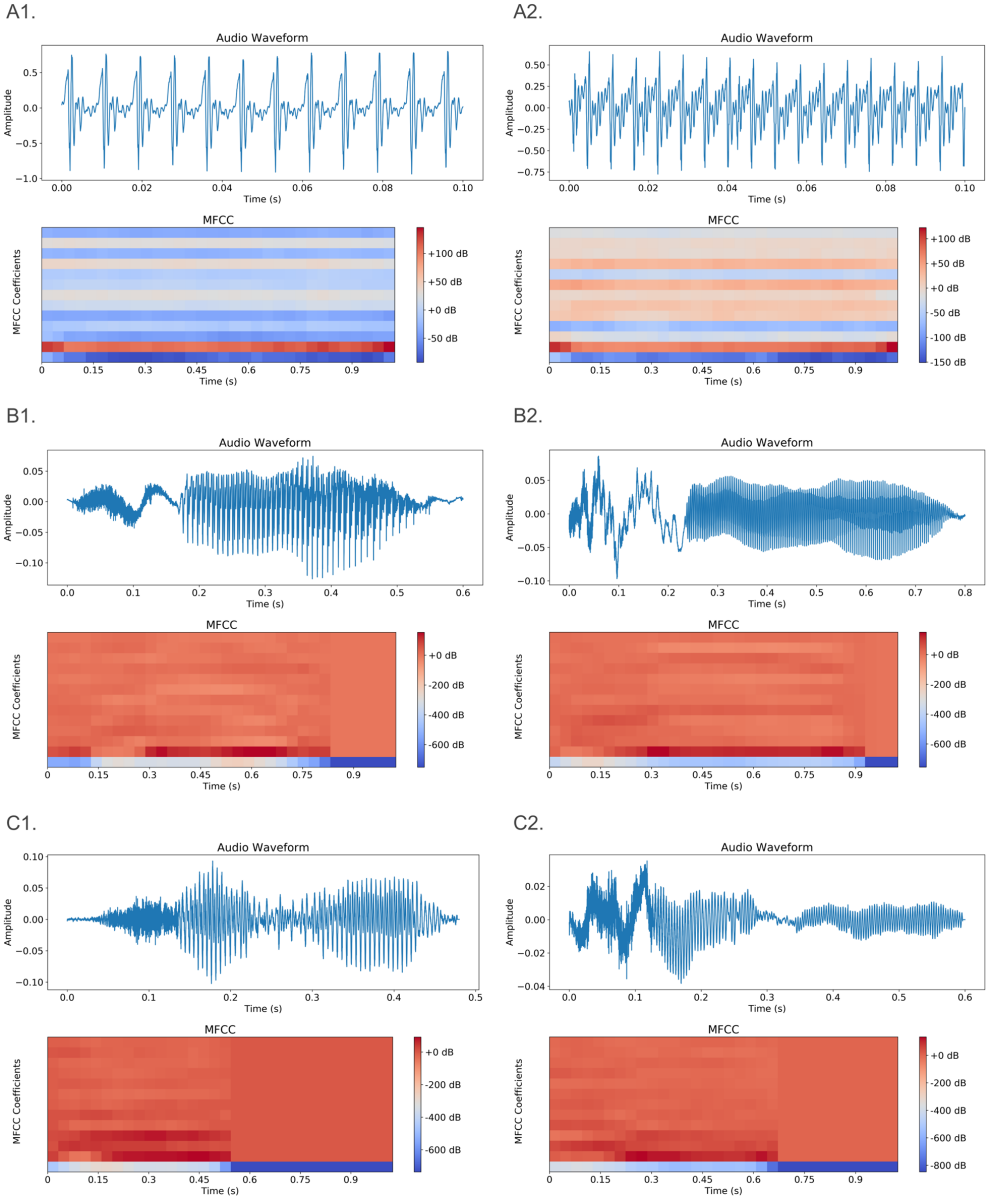
Comparison of audio wave and MFCCs of balanced and unbalanced individuals. A1 and A2 show audio wave form (top) and MFCC plot (bottom) of 0.1 s sustained [a] sound of balanced (left) and unbalanced (right) constitution types. B1 and B2 show audio wave form (top) and MFCC plot (bottom) of full [che] sound of balanced (left) and unbalanced (right) constitution types. C1 and C2 show audio wave form (top) and MFCC plot (bottom) of full [ji] sound of balanced (left) and unbalanced (right) constitution types. The visual differences are too subtle for human perception

The visual differences between the two types are noticeable, but discerning the exact differences remains challenging (Fig. 4). This necessitates the application of deep learning methodologies.

### Training accuracy

**Fig. 5:**
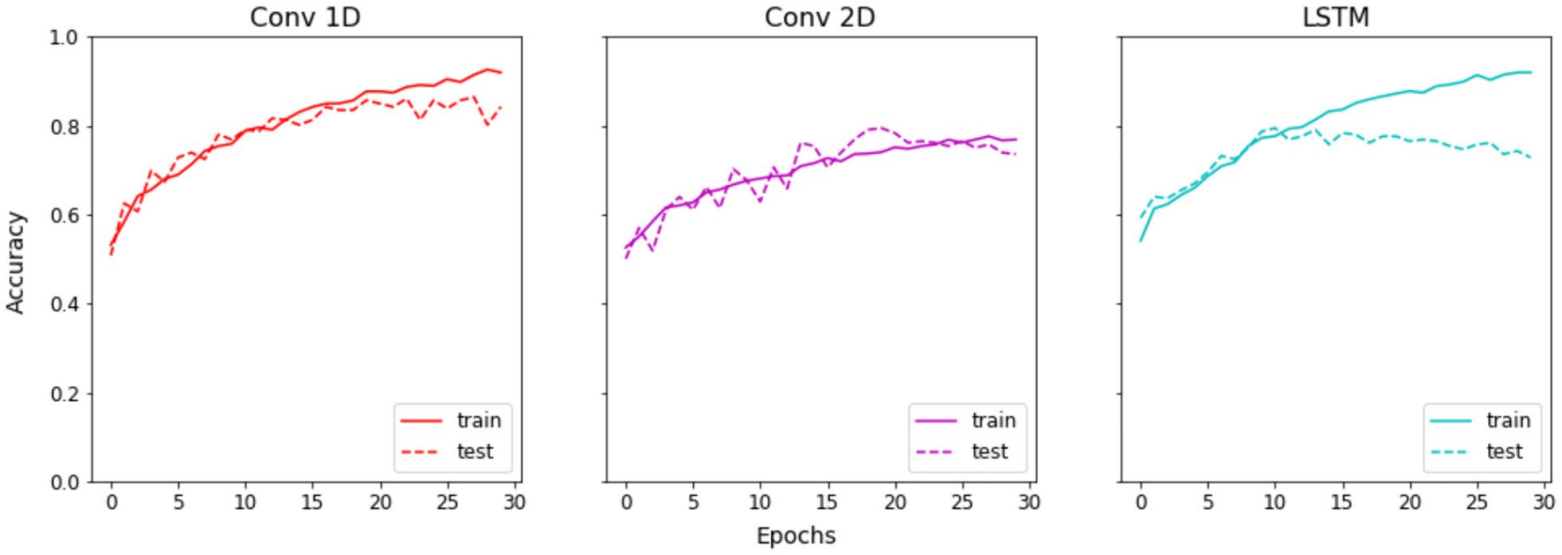
Deep learning model accuray plots

**Fig. 6:**
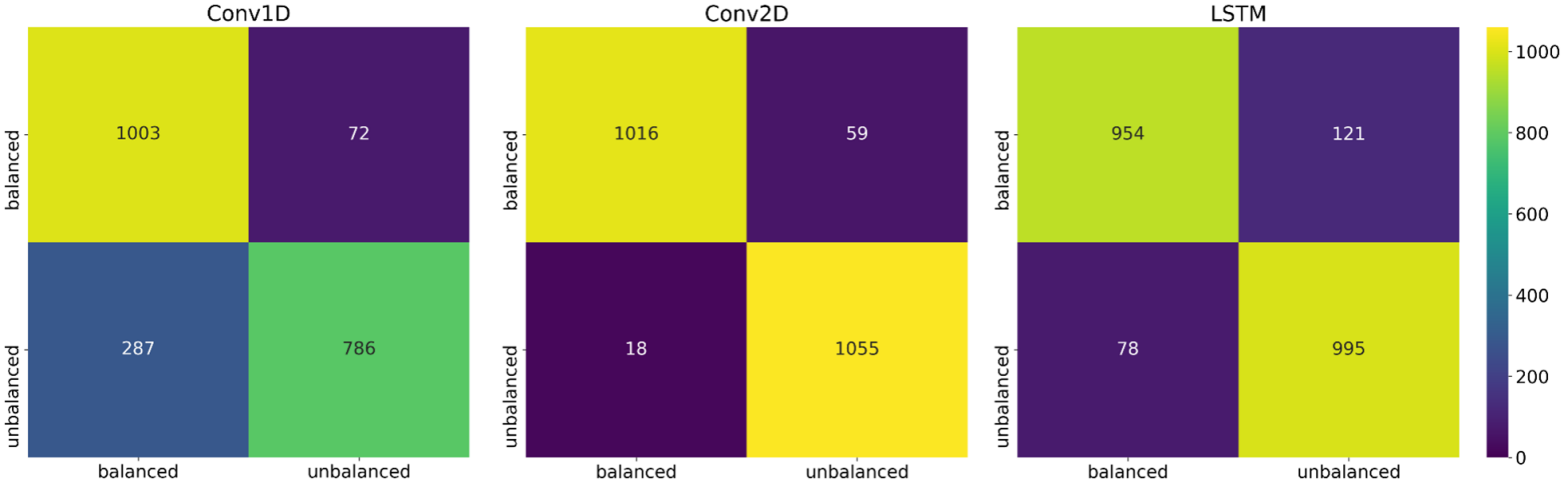
Confusion matrices for Conv1D, Conv2D, and LSTM models

The Conv1D model achieved a training accuracy of 91.91% and a validation accuracy of 84.19% at epoch 30 (Fig. 5). The Conv2D model attained a training accuracy of 96.19% and a validation accuracy of 84.93% at epoch 30, with both accuracies being comparable and indicating no signs of overfitting (Fig. 5). The LSTM model exhibited a training accuracy of 92.79% and a validation accuracy of 87.13% at epoch 30. However, the training and validation accuracies of the LSTM model began to diverge at epoch 10, suggesting the onset of slight overfitting (Fig. 5). Confusion matrices for all three models are shown in Fig. 6.

### Model performance metrics

The Conv1D model correctly predicts the class (balanced or unbalanced) for 83.3% of the instances. When it predicts an instance as balanced, it is correct 77.8% of the time, indicating a moderate level of precision. It correctly identifies 93.3% of the actual balanced instances. This indicates that the model is very good at identifying balanced instances. It has a good balance between precision and recall for the balanced class. It correctly identifies 73.2% of the actual unbalanced instances, which is relatively low compared to its recall. This indicates a higher rate of false positives for unbalanced instances. The Conv1D model shows good recall but has a lower precision and specificity, indicating that it tends to predict more false positives, especially for the unbalanced class. This results in a lower overall accuracy and F1 score compared to the previous model.

The Conv2D model correctly predicts the class (balanced or unbalanced) for 96.4% of the instances. When it predicts an instance as balanced, it is correct 98.3% of the time. It correctly identifies 94.5% of the actual balanced instances. It correctly identifies 98.3% of the actual unbalanced instances. It has a good balance between precision and recall for the balanced class. The Conv2D model performs very well in distinguishing between balanced and unbalanced instances, with high precision, recall, and accuracy.

The LSTM model correctly predicts the class (balanced or unbalanced) for 90.7% of the instances, indicating a high level of overall performance. When it predicts an instance as balanced, it is correct 92.4% of the time. This indicates a high level of precision with relatively few false positives. It correctly identifies 88.7% of the actual balanced instances, indicating a good but not perfect ability to capture all balanced instances. It has a strong balance between precision and recall for the balanced class, suggesting good overall performance in classifying balanced instances. It correctly identifies 92.7% of the actual unbalanced instances, indicating a high ability to capture unbalanced instances with relatively few false positives. The LSTM model demonstrates a strong performance, with high precision, accuracy, and specificity. Although its recall is slightly lower than that of the Conv1D model, it still maintains a good balance between precision and recall, as indicated by its high F1 score. This suggests that the LSTM model is effective in classifying both balanced and unbalanced instances, with a particularly strong performance in reducing false positives.

**Fig. 7:**
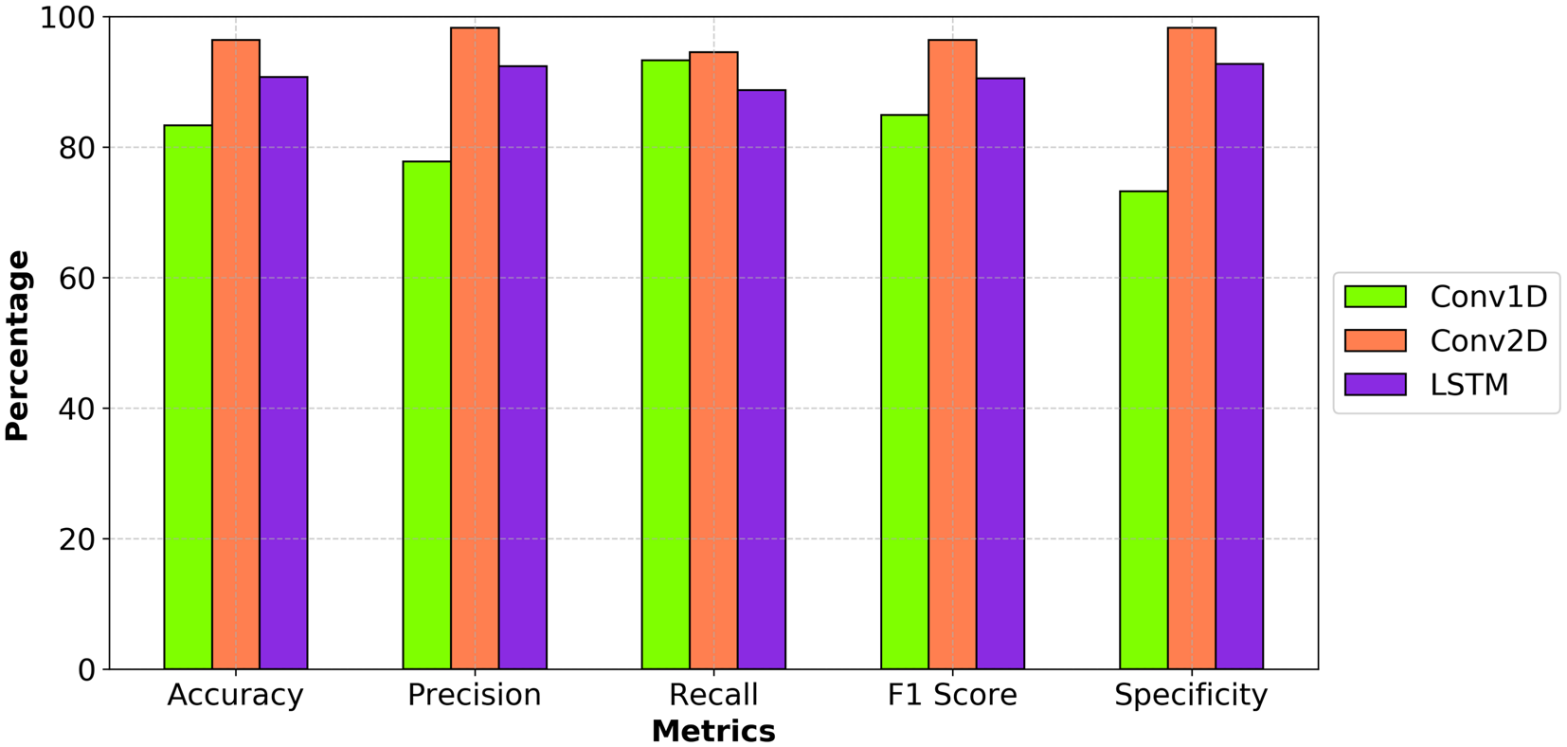
Model metrics comparison between Conv1D, Conv2D, and LSTM

According to model metrics of accuracy, precision, recall, F1 score, and specificity, Conv2D performs better than Conv1D and LSTM in binary classification of balanced and unbalanced constitution types using speech audio (Fig. 7).

### Area under the receiver operating characteristic

**Fig. 8:**
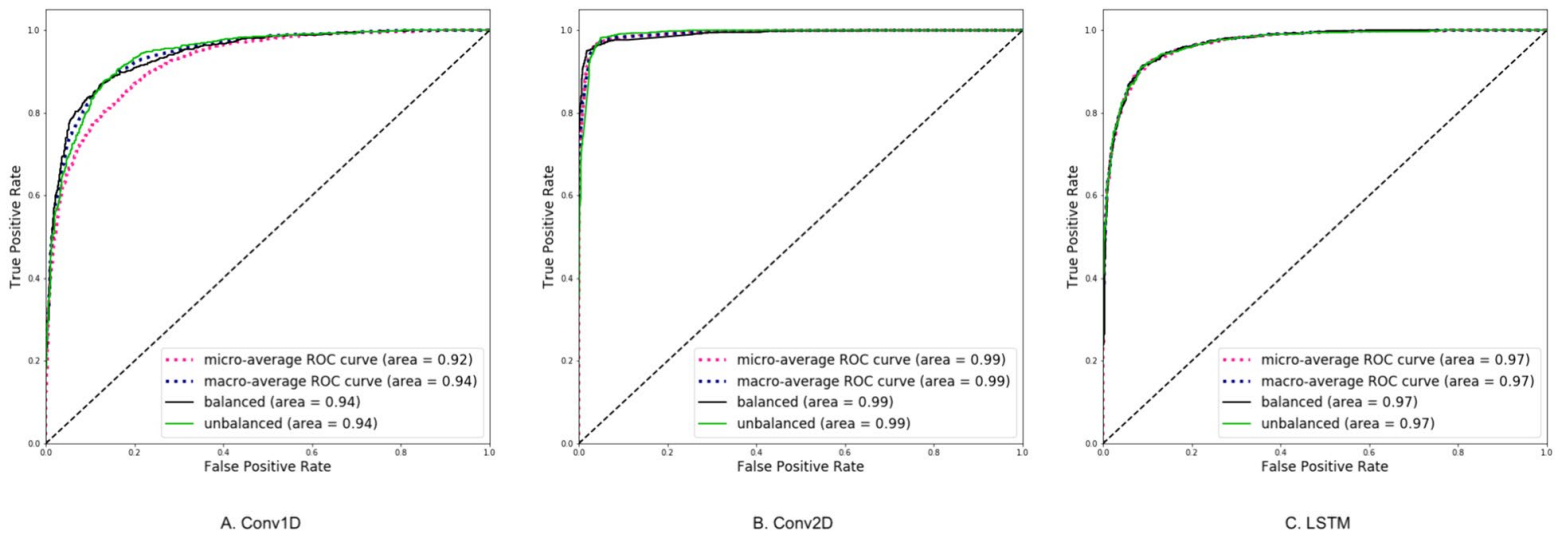
Area under the the receiver operating characteristic

The Conv1D model achieved micro-average and macro-average ROC curves with areas under the curve (AUC) of 0.92 and 0.94, respectively, for both balanced and unbalanced categories. The Conv2D model exhibited micro-average and macro-average ROC curves with AUC values of 0.99, while the LSTM model demonstrated micro-average and macro-average ROC curves with AUC values of 0.97 (Fig. 8).

### Saliency map of MFCCs

**Fig. 9:**
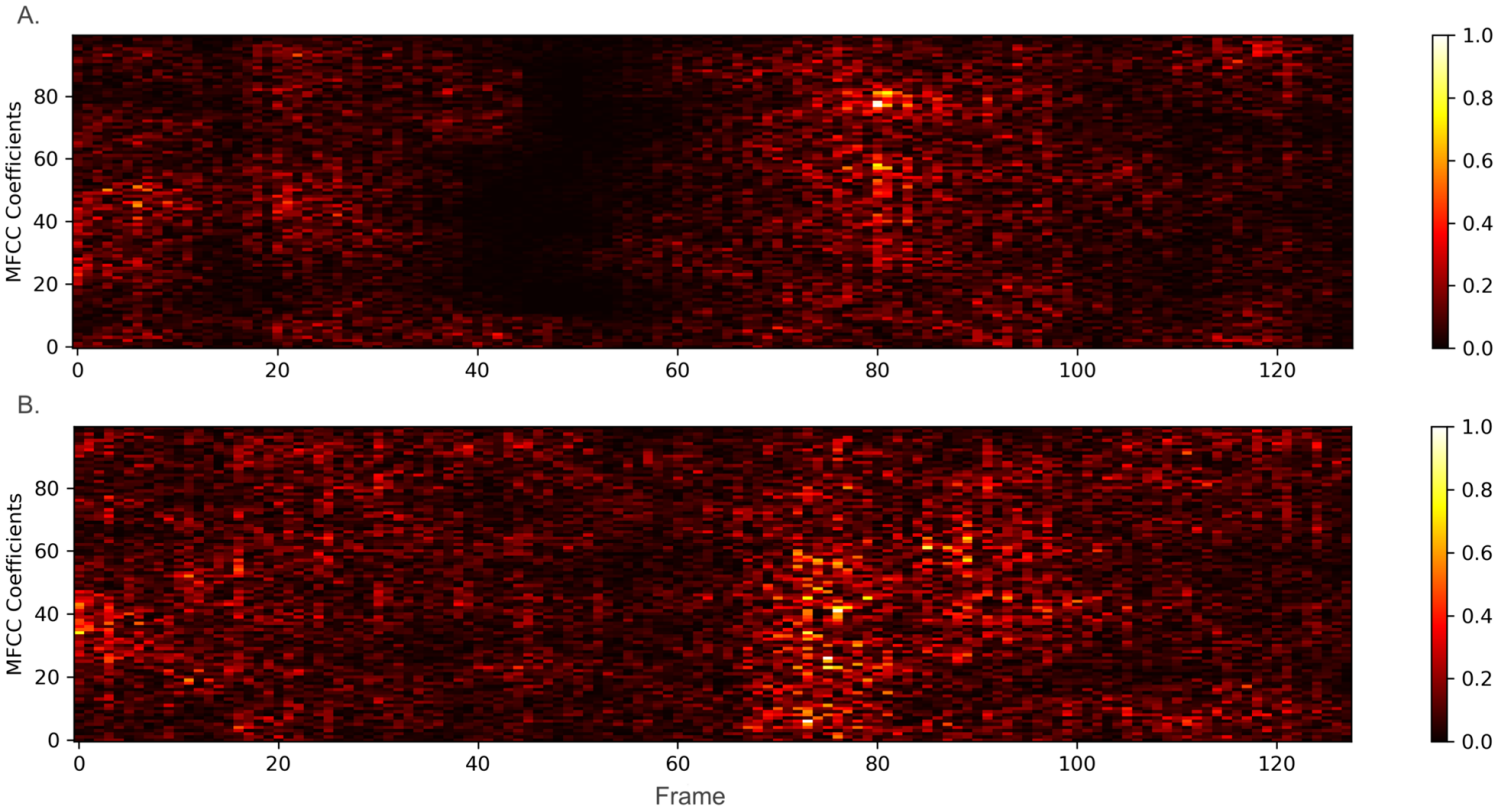
Conv2D model saliency map of MFCC coefficients. **A** balanced constitution type and **B** unblanced constitution type. Bright spots highlight the areas or MFCC features of the input that have the greatest impact on the model's output

The best performing model, Conv2D, is used to generate saliency maps (Fig. 9).

Conv2D model MFCC coefficients saliency map corresponds to speech audio of individuals of balanced and unbalanced constitution type. Conv2D correctly classifies the two types and the saliency maps reveal that different areas of the input data appear to be relevant for its classification decision. However, it is not possible to derive any deeper insights about the classification strategy of the model based on these visual explanations.

## Discussion

Mel-frequency cepstral coefficients of speech audio served as a valuable feature for differentiating health statuses in the current study. MFCCs play a crucial role in capturing and representing the characteristics of human speech [26], enabling the identification of changes in speech patterns that may indicate underlying health conditions, such as neurological disorders [27–29] or respiratory problems [30–32]. Beyond speech, MFCCs are also effective for analyzing different types of audio signals, including cough sounds [19, 31] or breathing patterns [19, 32], providing valuable insights into respiratory health. Their compact yet robust representation of audio signals makes them ideal for dimensionality reduction, simplifying feature extraction and facilitating subsequent analysis and classification tasks [33, 34]. By feeding MFCCs into machine learning algorithms, it becomes possible to train systems to recognize patterns associated with specific health conditions, enabling non-invasive and low-cost health monitoring solutions that offer real-time feedback for timely intervention or alerts in case of abnormal health conditions.

Both Conv1D and Conv2D are types of convolutional neural networks (CNNs), but they are used for different types of data and have distinct applications. Conv1D is primarily used for sequence data or time-series data. This includes applications such as audio signal processing, natural language processing, and any other domain where data is arranged in a sequential format. In Conv1D, the convolution operation slides a one-dimensional filter over the input data, capturing patterns and features along the temporal or sequential dimension [35]. Conv2D is commonly used for image and video data, where the data is arranged in two-dimensional grids (height and width). This type of CNN applies two-dimensional filters to the input, capturing spatial features such as edges, textures, and other patterns within the image. Unlike audio waves, which are one-dimensional, MFCCs are two-dimensional, like images, making them a good candidate for Conv2D networks. Conv1D and Conv2D both leverage the principles of convolution, pooling, and hierarchical feature extraction.

The Conv1D model demonstrated a robust training accuracy of 91.91% and a validation accuracy of 84.19% at epoch 30. This discrepancy between training and validation accuracy, although present, does not suggest significant overfitting, indicating that the Conv1D model generalizes reasonably well to unseen data.

The Conv2D model exhibited a higher training accuracy of 96.19% and a validation accuracy of 84.93% at epoch 30. The minimal gap between these accuracies implies that the Conv2D model also generalizes effectively without overfitting, despite its higher complexity compared to the Conv1D model. This suggests that the Conv2D model is more capable of capturing intricate patterns in the data, which is reflected in its superior training accuracy.

In contrast, the LSTM model achieved a training accuracy of 92.79% and a validation accuracy of 87.13% at epoch 30. While these accuracies are commendable, the divergence observed between training and validation accuracies beginning at epoch 10 suggests that the LSTM model is prone to overfitting. This early divergence highlights the model's sensitivity to the training data, which could be due to its recurrent nature and ability to capture temporal dependencies, leading to an overfitting tendency when exposed to the same patterns repeatedly.

The Conv1D model exhibited commendable performance with micro-average and macro-average ROC curves, achieving areas under the curve (AUC) of 0.92 and 0.94, respectively. This indicates that the model effectively discriminates between balanced and unbalanced constitution types across individual and class-wise evaluations. Notably, the AUC values for both balanced and unbalanced categories were consistent at 0.94, suggesting robustness in classification across different constitution types.

The Conv2D model demonstrated superior performance compared to Conv1D, as evidenced by significantly higher AUC values for both micro-average and macro-average ROC curves, with scores of 0.99. This indicates that the Conv2D model achieved near-perfect discrimination between balanced and unbalanced constitution types, showcasing its ability to capture intricate spatial patterns within the data. Similarly, consistent AUC values of 0.99 were observed for both balanced and unbalanced categories, reaffirming the model's effectiveness across different constitution types.

The LSTM model also exhibited strong classification performance, with micro-average and macro-average ROC curves achieving AUC values of 0.97. This indicates that the LSTM model effectively captures temporal dependencies in the data and discriminates between balanced and unbalanced constitution types. Consistent AUC values of 0.97 were observed for both balanced and unbalanced categories, suggesting the model's stability and reliability across different constitution types.

Micro-average ROC curve is calculated by summing up the true positives (TP), false positives (FP), true negatives (TN), and false negatives (FN) for each class, and then using these totals to calculate the overall TP, FP, TN, and FN rates. The micro-average ROC curve is sensitive to class imbalance, as it gives more weight to the majority class. Macro-average ROC curve is calculated by first calculating the TP, FP, TN, and FN rates for each class separately, and then averaging these rates across all classes. The macro-average ROC curve is less sensitive to class imbalance, as it gives equal weight to each class. Since the balanced and unbalanced types are mostly balanced, the two curves are closely shadow each other.

While the Conv1D and Conv2D models exhibit strong generalization capabilities, the LSTM model, despite its high performance, requires careful regularization to mitigate overfitting. Future work will explore techniques such as dropout, early stopping, or data augmentation to enhance the LSTM model's robustness. Additionally, fine-tuning hyperparameters and incorporating cross-validation could further balance the training and validation performance, ensuring the model's applicability to real-world data.

The training and validation accuracy results complemented the ROC curve findings, indicating the models’ ability to generalize to unseen data. Despite slight variations in training and validation accuracies, all models demonstrated robust performance without signs of overfitting, ensuring their reliability in real-world applications.

These findings underscore the potential of Conv1D, Conv2D, and LSTM models in accurately classifying health status types using human speech audio. While the Conv2D model exhibited the highest discriminatory power, all three architectures displayed promising performance, providing valuable insights into the classification of balanced and unbalanced constitution types.

### Comparisons between traditional voice quality parameters and MFCCs

Traditional voice quality parameters include fundamental frequency (F0), jitter, shimmer, harmonics-to-noise ratio (HNR), cepstral peak prominence (CPP), and maximum phonation time (MPT). The fundamental frequency measures the basic pitch of the voice and helps identify abnormal pitch levels, pitch variability, and vocal fold vibration issues. Jitter measures cycle-to-cycle frequency variation, indicating frequency stability, with higher values suggesting potential vocal pathologies. Shimmer measures cycle-to-cycle amplitude variation, indicating amplitude stability, with higher values suggesting potential vocal pathologies. HNR measures the ratio of harmonic sound to noise, where lower values indicate a breathy or rough voice. CPP measures the prominence of the cepstral peak, with higher values indicating clearer, more periodic voice signals. MPT measures the duration of sustained phonation, where reduced MPT indicates respiratory or vocal fold inefficiencies.

MFCCs represent the short-term power spectrum of an audio signal. They are computed by taking the Fourier transform of a windowed signal, mapping the powers of the spectrum to the Mel scale, taking the logarithm of the powers, and then performing the discrete cosine transform. MFCCs capture the spectral envelope of the voice, which includes important information about the vocal tract's shape and the characteristics of the sound produced. By analyzing the distribution of energy across different frequency bands, MFCCs provide information about the timbre of the voice, which is related to voice quality. They are useful in distinguishing between different speakers and identifying changes in voice quality over time, making them valuable for tasks such as emotion detection or speaker verification. MFCCs provide a broader spectral analysis that encompasses many aspects of the sound signal, useful for pattern recognition but less specific to clinical voice quality measures. The deep learning model results demonstrated that MFCCs are effective features for health status classification with high accuracy.

Unlike traditional voice quality parameters, MFCCs are not directly interpretable in clinical terms like jitter or shimmer. They are more abstract and require machine learning algorithms to extract meaningful information related to voice quality. Nevertheless, saliency maps highlight regions of importance on MFCCs. However, they do not provide detailed explanations of why these regions are significant or which features within these regions the model focuses on. They offer a simplified view of the model's behavior, often failing to capture interactions between features, which can lead to an incomplete understanding of the decision-making process. Additionally, the visual representation of saliency maps for audio data may not always be intuitive for human interpretation, especially for those without expertise in audio signal processing.

## Conclusions

The above models demand careful hyperparameter tuning to achieve optimal model performance. Manually selecting the best set of hyperparameters is a difficult and time- and resource-consuming task. The process of evaluating and adjusting the impact of architectural choices on model performance is iterative and requires careful consideration of both the classification task and dataset characteristics. Yet, the deep learning classification of human speech audio for health status classification using body constitution types yielded promising results across Conv1D, Conv2D, and LSTM models. Through the analysis of micro-average and macro-average ROC curves, as well as training and validation accuracy metrics, all models demonstrated robust performance in distinguishing between balanced and unbalanced constitution types. While the Conv2D model exhibited the highest discriminatory power, achieving near-perfect classification accuracy, the Conv1D and LSTM models also showcased impressive capabilities. Importantly, the consistency in model performance across different architectures underscores their reliability and generalizability in real-world applications.

These findings highlight the potential of deep learning approaches in using MFCCs for constitution type classification, paving the way for further research and advancements in personalized medicine and healthcare decision-making. This cost-effective, and non-invasive approach holds promise for developing personalized healthcare strategies that target individuals at risk of developing more serious health conditions, ultimately promoting early intervention and improved health outcomes. The personalized prevention strategy spearheaded by the current research has profound implications for global healthcare. Moving forward, leveraging ensemble methods or hybrid architectures could further enhance classification performance and address potential limitations of individual models. Additionally, continued research into feature engineering may optimize model performance and contribute to advancements in constitution type classification.

The current study employed speech audio and deep learning techniques to discern between two types of constitution: balanced and unbalanced, serving as proxies for health and subhealth respectively. Further research should aim to assess the applicability of these models in populations where Chinese is not the primary language. Additionally, future efforts should focus on refining the classification of the eight unbalanced types and mixed unbalanced types. This task is particularly challenging due to the complex and diverse nature of mixed types, making multi-label classification more demanding. Additionally, future work shall aim to gain a deeper understanding of the varied cepstral characteristics, identify the most influential features for classification, and elucidate their connection to clinical voice quality.

## Data Availability

The datasets generated and/or analysed during the current study are not publicly available because human voice data are biometric in nature and unable to be deidentified but are available from the corresponding author on reasonable request and upon ethics approval of Ethics Committee of Beijing University of Chinese Medicine.

## Abbreviations

TCM: Traditional Chinese Medicine
BC: Balanced constitution
QDC: Qi deficiency constitution
YDC: Yang deficiency constitution
YnDC: Yin deficiency constitution
PDC: Phelgm-dampness constitution
DHC: Dampness-heat constitution
BSC: Blood stasis constitution
QSC: Qi stagnation constitution
SDC: Special diathesis constitution
DCT: Discrete cosine transform
ROC: Receiver operating characteristic
AUC: The area under the ROC curve
CNN: Convolutional neural network
LSTM: Long short-term memory
Conv1D: 1-Dimensional convolution
Conv2D: 2-Dimensional convolution
MFCCs: Mel-frequency cepstral coefficients
TP: True positives
FP: False positives
TN: True negatives
FN: False negatives
HNR: Harmonics-to-noise ratio
CPP: Cepstral peak prominence
MPT: Maximum phonation time
